# Pulmonary interstitial glycogenosis in two neonates: Early recognition and use of corticosteroids^☆^

**DOI:** 10.1016/j.rmcr.2024.101990

**Published:** 2024-02-01

**Authors:** Eric Hamberger, Yolanda Yu, Hyo-Jung Choi

**Affiliations:** a Department of Pediatrics, Division of Pediatric Pulmonology and Sleep Medicine, University of California Los Angeles, Los Angeles, CA, USA; b Department of Pediatrics, Division of Neonatology and Developmental Biology, University of California Los Angeles, Los Angeles, CA, USA

## Abstract

Pulmonary interstitial glycogenosis (PIG) is known to be associated with a wide variety of congenital conditions, though the extent to which PIG contributes to clinical presentation and outcomes in infants remains controversial. We describe two cases of infants with congenital anomalies and respiratory distress at birth who were diagnosed with PIG with differing clinical courses and response to methylprednisolone therapy. These cases highlight the importance of improved recognition of PIG and uncertainties about which patients may benefit from treatment.

## 1 Introduction

Children's Interstitial and Diffuse Lung Diseases (chILD) comprise over 200 rare chronic respiratory diseases in children [1,2]. Pulmonary interstitial glycogenosis (PIG) is a rare form of chILD first described in 2002, based upon the presence of glycogen within interstitial mesenchymal cells [3,4]. Infants with PIG typically present with respiratory distress and hypoxemia at or shortly after birth [3,5]. The disorder is often found in association with other cardiopulmonary disorders including structural heart disease, pulmonary hypertension, alveolar simplification, and extrapulmonary diseases such as Trisomy 21, Noonan syndrome, and inborn errors of metabolism [[4], [5], [6], [7]].

CT findings in PIG may show diffuse ground glass opacities and cystic lucencies. Clinical and radiologic findings often overlap with other clinical entities presenting with neonatal respiratory distress, thus lung biopsy is required for diagnosis [8]. Histological findings characteristic of PIG include thickening of the alveolar interstitium with increased cellularity of mesenchymal cells and cytoplasmic glycogen with minimal or no intracellular glycogen within type II alveolar epithelial cells [3,4,9,10]. Alveolar simplification and pulmonary arterial wall thickening are also associated with this diagnosis [8].

There are no clinical consensus guidelines for the treatment of PIG, although systemic corticosteroid use is reported in most patients [4]. Disease severity differs greatly from patient to patient due to varying degrees of lung involvement, associated comorbidities, and early success of medical interventions and ventilatory support. As a result, outcomes can range from self-limited disease to rapid progression and death [2,11].

The cases of PIG presneted in this report shed light on a larger pattern of larger pattern of underrecognition due to provider hesitancy toward lung biopsy. Further, associated comorbidities such as pulmonary hypertension, structural heart disease, and absence of known genetic markers or pathognomonic radiographic findings may obscure recognition of this and other disease entities in the neonatal period. This report emphasizes a lower threshold for open lung biopsy, especially in infants undergoing thoracic surgical repair, to inform potentially life-saving medical management, in this case the use of systemic steroids. Greater recognition and reporting will also create expanded cohorts and enhance the strength of future studies within these populations.

## 2 Methods

Clinical history, laboratory findings, imaging studies, genetic testing, and pathology findings were obtained from the electronic medical records. This case series was written under the Institutional Review Board and Privacy Board approved protocols of the University of California Los Angeles. Parental written permissions were obtained.

## 3 Case presentations

Patient 1 was born at 37 weeks 5 days gestation with hypoxemia and respiratory distress. He was born by spontaneous vaginal delivery with Apgar scores of 8 and 9 (1 and 5 minutes, respectively), however shortly thereafter was noted to have low SpO2 without significant improvement with supplemental oxygen. He was subsequently intubated and mechanically ventilated. Transthoracic echocardiogram demonstrated hypoplastic left heart syndrome with mitral atresia, transverse aortic arch coarctation, ventricular septal defect, and mixed partial anomalous pulmonary venous return. Cardiac catheterization following his cardiac repair revealed lymphangiectasia. He underwent surgical repair by Norwood Procedure with a 3.5 mm Blalock-Taussig shunt and atrial septectomy.

Postoperative course was prolonged by recurrent chylous effusions requiring chest tube placement, recurrent left apical pneumothorax, and pulmonary edema requiring prolonged mechanical ventilation. He was treated with a course of IV methylprednisolone 1 mg/kg twice daily for 8 days.

**Fig. 1a, 1b, 1c:**
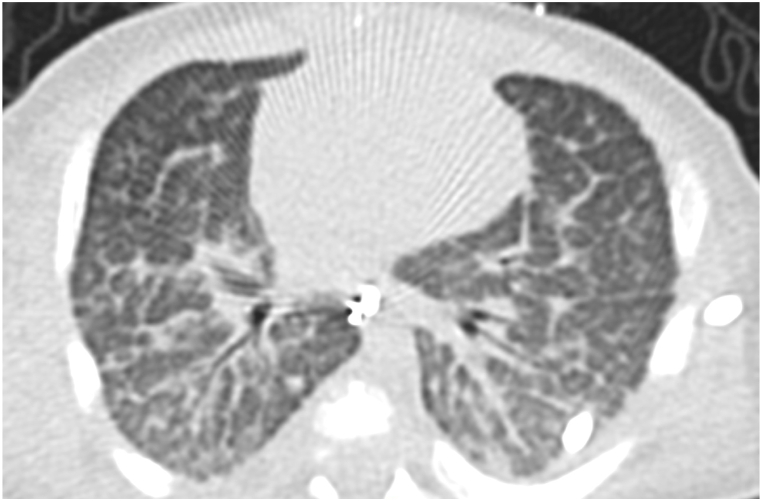

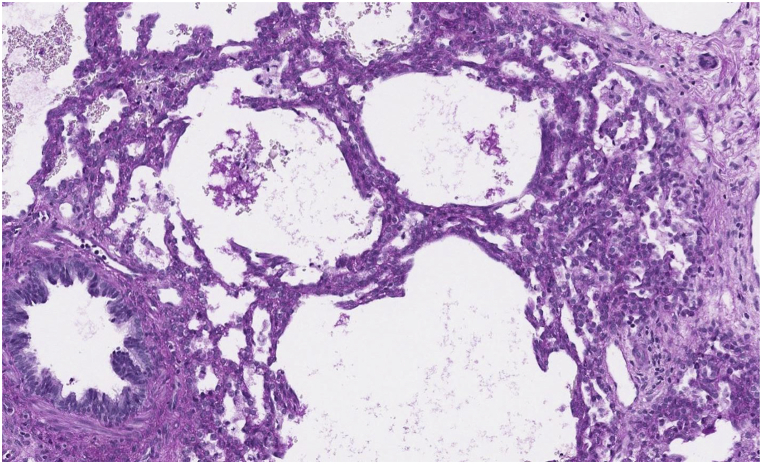

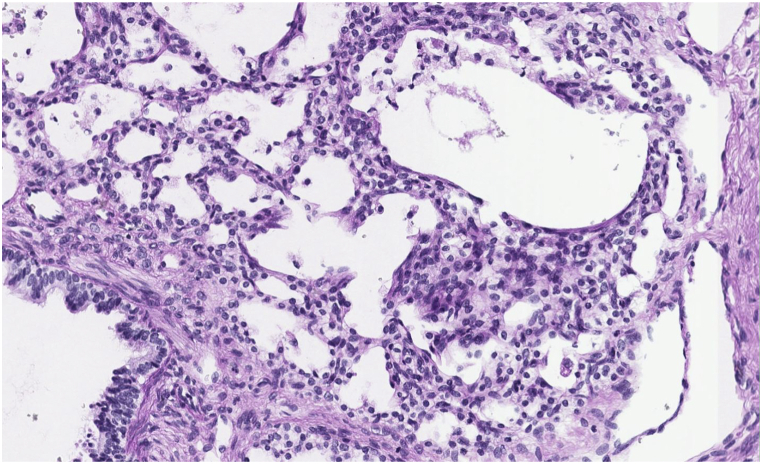
CT of the chest (bases) shows diffuse interstitial opacities. Interstitium is thickened and mildly irregular, more prominent in the dependent regions (1a). Periodic acid-Schiff stain (without and with diastase) confirms glycogen within interstitial mesenchymal cells (1b, 1c, respectively). Interstitial glycogenosis involves more than 30 % of the alveolar septa.

**Fig. 2a, 2b, and 2c:**
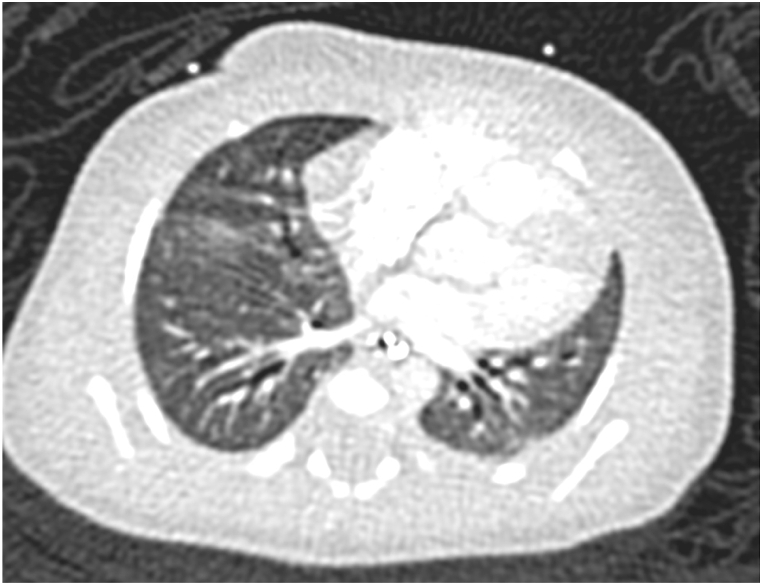

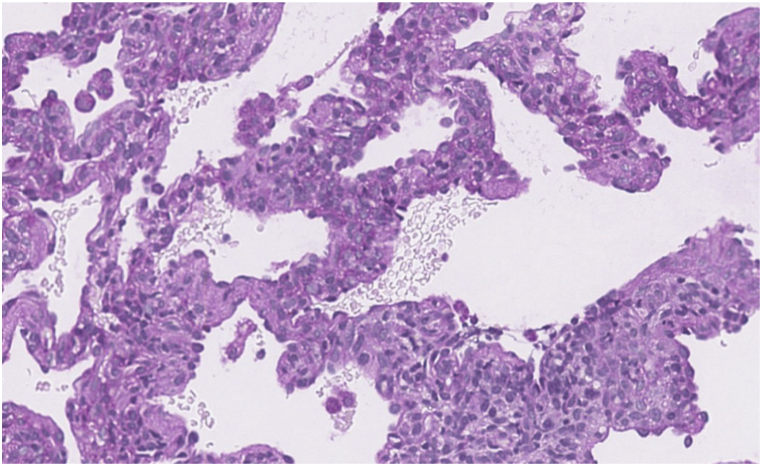

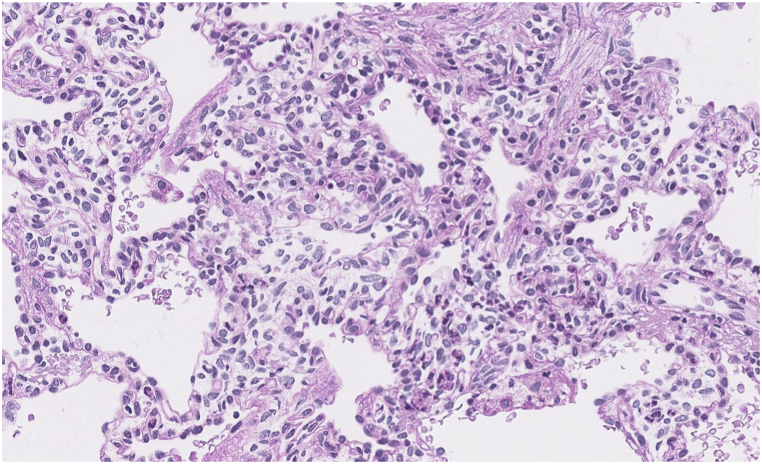
CT of the lung bases shows low lung volumes with diffuse interstitial thickening. Right hemidiaphragm is elevated (2a). Periodic acid-Schiff stain (without and with diastase) confirms glycogen within interstitial mesenchymal cells (2b, 2c, respectively). In some biopsy profiles it involves 60–70 % of the area. In others, where the alveolar simplification and enlargement are more prominent, PIG involves 20–30 % of the area.

CT scan of the chest was notable for diffuse bilateral interstitial opacities most prominent near the lung bases, with thickened interstitium and mild lymphangiectasia (Fig. 1). Based on these findings, genetic testing for 111 neonatal causes of respiratory distress was sent, and did not identify any pathogenic variants known to cause disease. Concurrently, wedge biopsy of the right lower lobe analyzed by periodic acid-Schiff stain confirming the presence of glycogen within interstitial mesenchymal cells involving more than 30 % of the alveolar septa, suggestive of pulmonary interstitial glycogenosis (Fig. 2b, c). Following the diagnosis of PIG, he was treated with 3 days of IV methylprednisolone therapy of 10 mg/kg daily for 3 days. The infant was initially discharged home with supplemental oxygen, however he was readmitted and placed on mechanical ventilation following a viral infection, complicated by worsening pulmonary hypertension. Tracheostomy was placed for chronic mechanical ventilation and the patient was discharged under the care of his family.

Patient 2 was born at 39 weeks gestation by spontaneous vaginal delivery with poor respiratory effort and central cyanosis. Apgar scores at 1, 5, and 10 minutes were 1, 5, and 6, respectively. She was started on positive pressure ventilation at 1 min of life, then intubated and placed high frequency oscillatory ventilation. She was subsequently placed on synchronized intermittent mandatory ventilation. Chest radiograph showed marked elevation of the hemidiaphragms, hypoplastic lungs, and compression of the cardiomediastinal structures. Further evaluation by targeted ultrasound of the right chest demonstrated a right congenital diaphragmatic hernia with liver herniation. Cardiac workup determined a ventricular septal defect and pulmonary hypertension.

She underwent venoarterial extracorporeal membrane oxygenation (ECMO) cannulation on day of life 1 and was decannulated 10 days later. While on ECMO support, the infant underwent diagnostic thoracoscopy, exploratory laparotomy, right lung wedge biopsy, and repair of a right-sided congenital diaphragmatic hernia (CDH) via transabdominal approach on day of life 5. Initial CT scan prior to hernia repair showed elevation of the right hemidiaphragm with low lung volumes, and left sided mediastinal shift. CT scan following CDH repair showed improved pulmonary aeration, low lung volumes and extensive atelectasis with slight relative elevation of the right hemidiaphragm (Fig. 2a).

Tissue exam of right lung biopsy described alveolar growth abnormality with periodic acid-Schiff stains demonstrated abundant non-membrane bound, monoparticulate glycogen within mesenchymal cells estimated to involve more than 30 % of the area, as well as areas of alveolar simplification (Fig. 2b, c). Genetic testing (as in patient 1) was negative for any pathogenic variants of disease known to cause neonatal respiratory distress.

The infant was treated with 3 days of IV methylprednisolone therapy of 10 mg/kg daily for 3 days with an additional 9-day taper, and started inhaled budesonide 0.25mg twice daily. During the following two months, ventilator settings were decreased gradually, however after multiple failed extubation attempts, a tracheostomy was placed for long-term mechanical ventilation. At 3 months of life, she showed moderate clinical progress with significant improvement of pulmonary hypertension. She remains on around-the-clock mechanical ventilation and was discharged to a chronic care facility.

Patient characteristics, clinical presentations, clinical course, and outcomes are described in Table 1.

**Table 1:** Patient characteristics.

	Patient 1	Patient 2
**Sex**	Male	Male
**Race/Ethnicity**	Caucasian	Hispanic
**Delivery**	Spontaneous Vaginal	Spontaneous Vaginal
**Birth Weight**	3460g	2521g
**Gestational Age**	37 weeks 5 days	39 weeks
**Apgar scores (1 min, 5 min, 10 min)**	8, 9, 9	1, 5, 6
**Presentation at delivery**	Desaturation and increased respiratory effort at 1 minute	Central cyanosis at birth, minimal respiratory effort
**Interventions**	Intubated and placed on HFOV	Intubated and placed on HFOV; ECMO x10 days
**Associated comorbidities**	Hypoplastic Left Heart Syndrome, pulmonary hypertension	Right sided Congenital Diaphragmatic Hernia, pulmonary hypertension
**PIG Treatment**	IV methylprednisolone 1 mg/kg twice daily for 8 days (prior to diagnosis of PIG) IV methylprednisolone 10 mg/kg daily x3 days Inhaled budesonide 0.25 mg daily	IV methylprednisolone 10 mg/kg daily x3 days followed by 9 day taper Inhaled budesonide 0.25 mg daily
**Outcome at the time of last follow up**	Tracheostomy/Ventilator Dependence; Alive	Tracheostomy/Ventilator Dependence; Alive

*HFOV*: high-frequency oscillatory ventilation

*ECMO*: extracorporeal membrane oxygenation.

## 4 Discussion

*Clinical discussion:* We describe two cases of infants with congenital anomalies and pulmonary interstitial glycogenosis. To our knowledge, congenital diaphragmatic hernia and hypoplastic left heart syndrome have each been described only once and twice before, respectively, in association with PIG [4,11]. Each patient underwent surgical correction of the underlying anomaly with concurrent lung biopsy. Of note, genetic testing was obtained in both infants, and did not identify pathogenic variants known to cause neonatal respiratory distress. As there are currently no radiologic or genetic markers to confirm the diagnosis of PIG, both cases would have been undiagnosed had a biopsy not been obtained [12]. For this reason, we suspect PIG to be an underdiagnosed clinical entity, underscoring the importance of a comprehensive diagnostic approach in cases of suspected chILD, especially when clinical presentation is out of proportion to the severity of the associated condition. Analysis of these cases in the broader context of PIG is limited by differences in associated congenital anomaly, presence of pulmonary hypertension, and therapeutic approach.

*Brief review of literature:* Both CDH and HLHS have been described in previous case series, although congenital anomalies of the heart are commonly associated with PIG, with one single-center retrospective review reporting 63 % of patients with biopsy-confirmed diagnosis [4]. In the same study, one patient was found to have a congenital diaphragmatic hernia in addition to numerous structural heart defects and thus a more severe clinical course. Hypoplastic left heart syndrome has been described in at least two prior case series [4,11].

Classification of PIG is categorized by both distribution of lung disease and secondary association. With regard to disease distribution, PIG is characterized as either a patchy or diffuse process, with the former associated with disorders of alveolarization, pulmonary hypertension, and congenital lung malformations, and a wider distribution in prognosis [9]. By contrast, diffuse PIG is less commonly observed in association with other findings and prognosis is favorable [13]. In the case series by Liptzin et al. disease distribution was observed equally among infants with PIG, however, another multicenter review by Seidl et al. described a predominance of diffuse PIG-pattern [4,5]. Sample sizes in both reports were small (22 and 8, respectively) due to disease rarity. A larger review of 28 cases by Cutz et al. stratifies patients into four groups based on PIG with abnormalities of alveolar development, PIG with congenital heart disease and vasculopathy, PIG with prominent pulmonary neuroendocrine cell and vasculopathy, and PIG associated with congenital lung malformations [11].

Unlike other interstitial diseases of infancy such as disorders of surfactant dysregulation, excess molecular deposition in PIG is not thought to be a disorder of homeostasis, and may explain why a single gene mutation has not been identified [10,14]. Pathogenesis of PIG is hypothesized to be a disorder of lung maturation based on established knowledge of the presence of large amounts glycogen in the cytoplasm of epithelial cells lining airways and developing alveoli, and loss of glycogen later in lung maturation correlating to the production of surfactant [11].

An etiology of pulmonary maturational arrest in PIG is supported by several observational studies. Clinical improvement has been reported in response to systemic steroids resulting in accelerated alveolar remodeling and surfactant production [9,15,16]. One case report described rapid clinical improvement with reduced cell proliferation and apoptosis of mesenchymal cells in two specimens obtained before and after steroid administration, suggesting that alterations in tissue remodeling may play a role in both disease pathology and improvement with steroids [15]. While these improvements are promising, others acknowledge the negative consequences of corticosteroids on postnatal alveolarization and neurodevelopmental outcomes [13].

Both patients in this report received IV methylprednisolone based on prior case analyses rather than established clinical guidelines. In both patients, PIG was found on lung biopsy during surgical repair of associated congenital anomalies. Improved recognition of PIG would inform future studies on the effectiveness of systemic steroids.

## 5 Conclusion

• Pulmonary Interstitial Glycogenosis (PIG) is a form of children's interstitial lung disease (chiLD) characterized by the presence of glycogen within interstitial mesenchymal cells.
• The absence of distinctive genetic markers or specific radiologic indications necessitates a lung biopsy for diagnosing PIG, leading to potential underreporting of cases.
• Infants displaying signs of chILD, particularly those undergoing surgical correction for congenital abnormalities, should be considered for open lung biopsy with a lowered threshold if genetic or radiologic evidence is not identified.

## Funding/support

No funding was secured for this study.

## Role of funder/sponsor (if any)

Not applicable.

## Author contributors statement page

Drs Eric Hamberger, Yolanda Yu, and Hyo-Jung Choi collected data, drafted the initial manuscript, and critically reviewed and revised the manuscript.

All authors approved the final manuscript as submitted and agree to be accountable for all aspects of the work.

## CRediT authorship contribution statement

**Eric Hamberger:** Conceptualization, Data curation, Formal analysis, Methodology, Writing – original draft, Writing – review & editing. **Yolanda Yu:** Conceptualization, Data curation, Writing – original draft, Writing – review & editing. **Hyo-Jung Choi:** Conceptualization, Data curation, Writing – original draft.

## Declaration of competing interest

No honorarium, grant, or other form of payment was given to anyone to produce this manuscript. The authors declare no conflicts of interest.
